# The Lisbon Supramolecular Green Story: Mechanochemistry towards New Forms of Pharmaceuticals

**DOI:** 10.3390/molecules25112705

**Published:** 2020-06-11

**Authors:** João Luís Ferreira da Silva, M. Fátima Minas da Piedade, Vânia André, Sofia Domingos, Inês C. B. Martins, M. Teresa Duarte

**Affiliations:** 1 Centro de Química Estrutural, Instituto Superior Técnico, Universidade de Lisboa, Avenida Rovisco Pais, 1049-001 Lisboa, Portugal; joao.luis@ist.utl.pt (J.L.F.d.S.); mdpiedade@fc.ul.pt (M.F.M.d.P.); vaniandre@tecnico.ulisboa.pt (V.A.); sofiadomingos@ff.ulisboa.pt (S.D.); ines.martins@tecnico.ulisboa.pt (I.C.B.M.); 2 Departamento de Engenharia Química, Instituto Superior Técnico, Universidade de Lisboa, Avenida Rovisco Pais, 1049-001 Lisboa, Portugal; 3 Departamento de Química e Bioquímica, Faculdade de Ciencias, Universidade de Lisboa, 1649-016 Lisboa, Portugal; 4 Associação do Instituto Superior Técnico para a Investigação e Desenvolvimento (IST-ID), Av. Rovisco Pais, 1049-003 Lisboa, Portugal

**Keywords:** active pharmaceutical ingredient, mechanochemistry, supramolecular interactions, cocrystals, molecular salts

## Abstract

This short review presents and highlights the work performed by the Lisbon Group on the mechanochemical synthesis of active pharmaceutical ingredients (APIs) multicomponent compounds. Here, we show some of our most relevant contributions on the synthesis of supramolecular derivatives of well-known commercial used drugs and the corresponding improvement on their physicochemical properties. The study reflects, not only our pursuit of using crystal engineering principles for the search of supramolecular entities, but also our aim to correlate them with the desired properties. The work also covers our results on polymorphic screening and describes our proposed alternatives to induce and maintain specific polymorphic forms, and our approach to avoid polymorphism using APIs as ionic liquids. We want to stress that all the work was performed using mechanochemistry, a green advantageous synthetic technique.

## 1. Introduction

Launching new active pharmaceutical ingredients (APIs) in the market is expensive, time consuming, and requires the use of complex production processes. Alternatively, pharmaceutical companies started developing strategies to improve the efficacy of well-known APIs with the aim of enhancing their physicochemical properties (solubility, dissolution rate, and bioavailability) [1,2,3] without changing their pharmacological behavior. Several approaches have been employed over the last years to achieve this goal, including the preparation of API multicomponent crystal forms (co-crystals and salts) [4,5]. 

Crystal engineering is a powerful tool in the rationalization of the synthesis of these new crystalline forms, searching for molecules with adequate functional groups to establish stable synthons [6] through molecular recognition, mostly involving hydrogen bonds. Defined in 1988 by Gautam Desiraju as the understanding of intermolecular interactions in the context of crystal packing and the utilization of such an understanding in the design of new solids with desired physical and chemical properties [7], it is widely used to predict and analyze the effect of structural variation in the physicochemical properties of APIs and multicomponent API derivatives [8,9,10]. 

Although the vast majority of the APIs (95%) are marketed as solid crystalline dosage forms, for reasons of purity, thermal stability, and the manufacturing process, there is an increasing interest in developing amorphous pharmaceutical or room temperature ionic liquids (RTILs) [11]. These compounds can not only enhance the APIs’ solubility but also avoid their polymorphic transformations, which are responsible for affecting their physicochemical properties. In this review, we will consider polymorphs as compounds that must present the same chemical composition, therefore excluding solvates or hydrates [12].

Synthetic approaches employed to prepare new multicomponent forms of APIs usually involve solution techniques, as they allow a good control of the reaction parameters (e.g., rate and yield). However, the large amounts of solvent (approximately 85% of the chemicals utilized in the pharmaceutical industry) used, apart from increasing the cost of products, cause environmental pollution and can be harmful for patients [13]. Searching for an answer to these problems has led to an increase in the use of solid-state mechanochemical techniques in the preparation of API derivatives. These solvent-free processes are also used in the synthesis of organic, inorganic, and organometallic compounds, polymers, and, more recently, metal–organic frameworks [13,14,15,16,17,18,19]. Mechanochemistry is based on the energy transfer from mechanical force to promote chemical or structural transformations. It emerged as an alternative process for the synthesis of a 1:1 co-crystal of 4-aminobenzoic acid and 3,5-dinitrobenzoic acid, in 1989 in a seminal work of Etter and Frankenbach, by manually grinding the solids with a mortar and pestle [20].

Grinding not only produced considerably smaller amounts of waste (it can be solvent free) but also proved to be a more practical and efficient (short reaction time, low temperature, absence of solvent effects) technique for the synthesis of new compounds, particularly for the screening of new API derivatives [18]. Another factor in favor of mechanochemistry is its capability to perform syntheses of products that could not be obtained by solution processes [16].

The first major development of mechanochemistry was the introduction of automated shaker and planetary ball mills [21,22]. The improvement in the control of parameters, such as the frequency and milling time, both reflected in the energy transferred, resulted in an increase of the reaction reproducibility. They also contributed to a scale-up of the reactions (from 5 mL up to 25 L) [23], both in the quantities of reactants and in time, as well as an increase of the process safety. The technique evolved with the introduction of liquid-assisted grinding (LAG), involving the addition of small volumes of solvent (in the scale of μL) to the solid reagents [24]. The presence of these solvent drops does not substantially increase the amount of waste produced, so it remains a green technique, but it does provide an increase in the mobility of the reactants, therefore improving the kinetics of product formation and allowing for the synthesis of compounds that could not be obtained by neat grinding (NG) [5,19]. LAG allows a better control of polymorphism [15] than NG and also solves crystallization problems that result from the solubility of co-crystal components, due to the permanent saturation of the solution, which takes it into a region of the ternary diagram that favors co-crystals over the crystallization of starting materials [16,18,21].

In this short review, we will mainly focus on the results obtained by the Lisbon team on co-crystals and salts, using mechanochemistry.

## 2. Results

In the last 12 years, our group has been involved in the synthesis and structural characterization of API co-crystals and salts, using mechanochemistry as the primary synthetic technique. We will highlight here some of the results we obtained using different APIs, whose physicochemical properties we were able to improve. Drug polymorphism has also been screened in our lab, and more recently, we developed new ionic liquids bearing the API (API-IL) as the principal constituent, using yet again mechanochemistry. We also addressed the study of several other API multicomponent forms, such as the antibiotic 4-aminosalisilic acid [25,26], paracetamol [27]. gabapentin lactam [28], the anti-hipertensive perindopril erbumine [29], and bismuth salicylate [30] that we are not referring to here, as they were done in straight collaboration with other international labs or the Portuguese industry. In all these works we used mechanochemistry as a synthetic technique, which we are also applying in the group in another context, which is that of API coordination networks [31].

### 2.1. Gabapentin

#### 2.1.1. Polymorphic Screening and Control

Gabapentin is a neuroleptic drug used for the prevention of seizures and for the treatment of mood disorders, anxiety, and tardive dyskinesia, as well as for the treatment of neuropathic pain and limb tremor, presenting a high solubility but with limited and variable bioavailability [32,33,34]. It is known in three anhydrous polymorphic forms (Form II, Form III, Form IV) [35,36,37], and a monohydrate form (Form I) [38,39]. 

**Figure 1:**
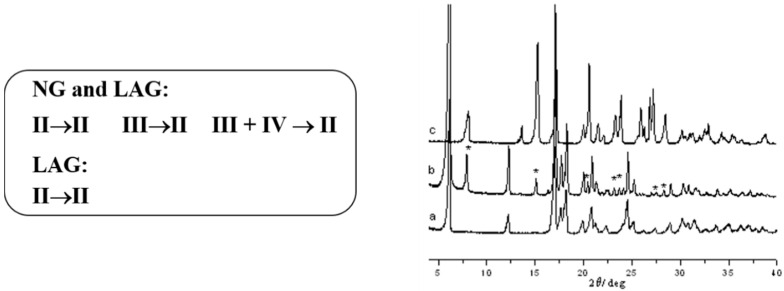
(**left**) Summary of the neat (NG) and liquid assisted grinding (LAG) polymorphic stability experiments; (**right**) XRPD patterns of (a) Form III, (b) Form III after grinding (stars indicate peaks of Form II) and (c) Form III after LAG using ethanol leading to complete conversion into Form II [37].

We conducted several studies to determine their thermal behavior, reactivity, and interconversion in different RH% conditions. In shelf conditions, 50%RH, Form II remains stable for 3 months, while the other forms evolve to Form II. At 100%RH all the forms change to Form I. Screening tests, using neat (NG) and liquid-assisted grinding (LAG) experiments, showed that Form II is the most stable form, while Forms III and IV convert into Form II. Upon grinding, Form II remains unchanged under the same conditions (Figure 1). Slurry tests using methanol always led to Form II, while in water the most stable is Form I, the hydrated form. We also demonstrated that Forms III and IV appear concomitantly and are impossible to isolate as pure in a bulk [37].

This experiment led us to conclude, like several other authors, that understanding, predicting, or controlling polymorphism is quite a challenge [40]. The importance of controlling API polymorphism is well known and it is a topic of high importance as 9 out of 10 marketed drugs are sold as solid dosage forms, preferably crystalline, due to their purity and thermal stability [41]. However, this is a problem as they are prone to polymorphic interconversion and it is well known that polymorphs’ physicochemical properties vary drastically [42].

Amidst the different factors influencing the crystallization process, the specific solvent–solute interactions are of major importance, and the solvent can be a determinant in the final polymorphic form [43]. These considerations led us to make use of RTILs, room temperature ionic liquids, as solvents to assess a possible control gabapentin polymorphic behavior [44].

**Figure 2:**
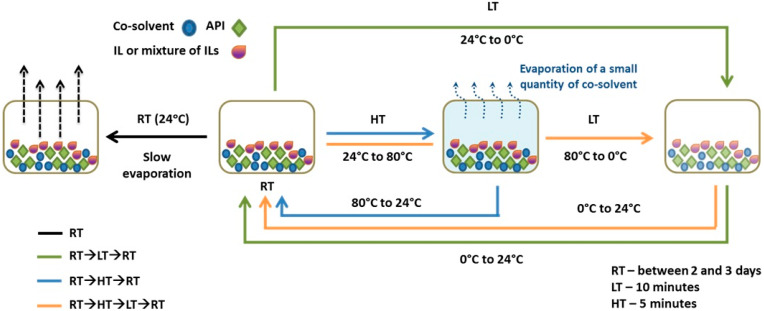
Schematic representation of the complete process followed to obtain GBP crystals. Conditions steps are represented with different colored arrows: RT (black); RT→HT→RT (green); RT→HT→RT (blue) and RT→HT→LT→RT (orange). RT, LT and HT stand for 24, 0, and 80 °C, respectively [44].

Using imidazolium-based RTILs either pure or as a mixture, we were able to induce mechanochemically the crystallization of the different polymorphic forms and isolate them as pure phases (Table 1), referring to temperature variations as seen in Figure 2.

So, for the first time, pure “bulk” GBP Form IV, a highly unstable polymorph, was isolated as well as Form III. These results were ascertained by single crystal structure determination, and bulk purity was confirmed by powder X-ray diffraction experiments. Molecular dynamics simulations clearly supported the results obtained.

**Figure 3:**
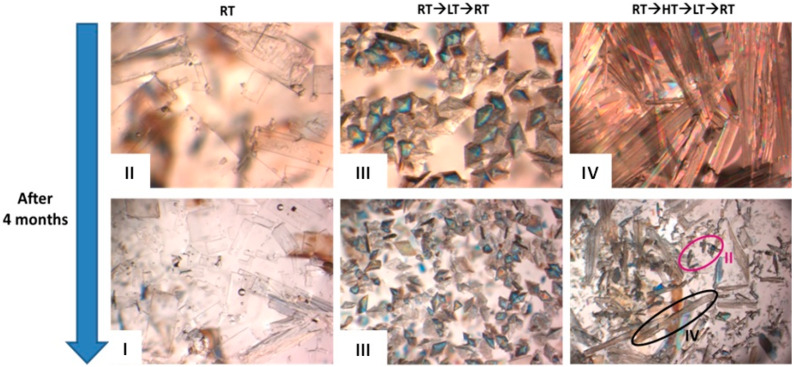
Crystal images of GBP polymorphic forms obtained in C_4_mimBF_4_+C_6_mimBF_4_ after crystallization and after 4 months [44]. Images obtained using an optical microscope.

However, besides inducing the preferred crystallization of a polymorphic form, it was also possible to conclude that as long as the different polymorphic forms were kept soaked in the RTILs mixtures, they remained stable over time for up to 4 months (Figure 3).

These results opened new perspectives in polymorphic-induced crystallization, so controlling the form’s appearance and maintaining it over time.

#### 2.1.2. Novel Multicomponent Crystal Forms and Ionic Liquids

**Figure 4:**
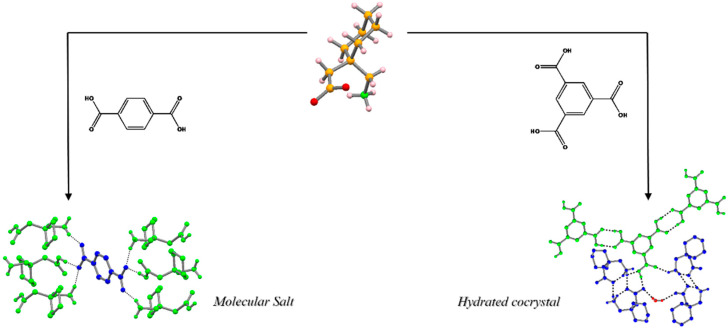
Schematic representation of the new multicomponent crystal forms of gabapentin with terephthalic and trimesic acids [45].

In the group we used gabapentin as a model case and, besides the polymorphic studies we presented, we also mechanochemically synthesized multicomponent crystal forms. Five new multicomponent crystal forms with isophthalic acid, phthalic acid, l-glutamine, terephthalic, and trimesic acids were prepared. The single crystal structures of the last two were determined and they were further studied by Differential Scanning Calorimetry (DSC), Thermogravimetric Analysis (TGA), Hot Stage Microscopy (HSM), and Infrared Spectroscopy (IR), and proved to be a hydrated co-crystal with trimesic acid and a molecular salt with terephthalic acid (Figure 4) [45]. The use of mechanochemistry presents a major advantage, especially with terephthalic acid, due to its very low solubility in water, which precluded a meaningful yield using the solution method. Both forms are based on carboxyl⋅⋅⋅carboxylate and amine⋅⋅⋅carboxylate synthons. The hydrate co-crystal with trimesic acid loses the water molecule in the 70–120 °C range and melts at 159 °C, while the molecular salt with terephthalic acid melts at 150 °C. For these forms, we also studied their pH stability; the trimesic acid co-crystal was shown to be stable to decomposition in the range of pH 3–8 while the form with terephthalic acid was only stable between pH 6 and 8. It is worth recalling that the co-crystal with trimesic acid is stable in a wider pH range than any of the gabapentin polymorphic forms [45]. 

Pursuing our studies with gabapentin and our work with polymorphism, again using mechanochemistry, we decided to check if we could avoid gabapentin polymorphism by using it as a liquid instead of a solid. We synthesized four new ionic liquids containing gabapentin, both as an anion and as a cation, which we called mechanoAPI-ILs (Active Pharmaceutical Ingredient-Ionic Liquids obtained mechanochemically) [46]. 

**Figure 5:**
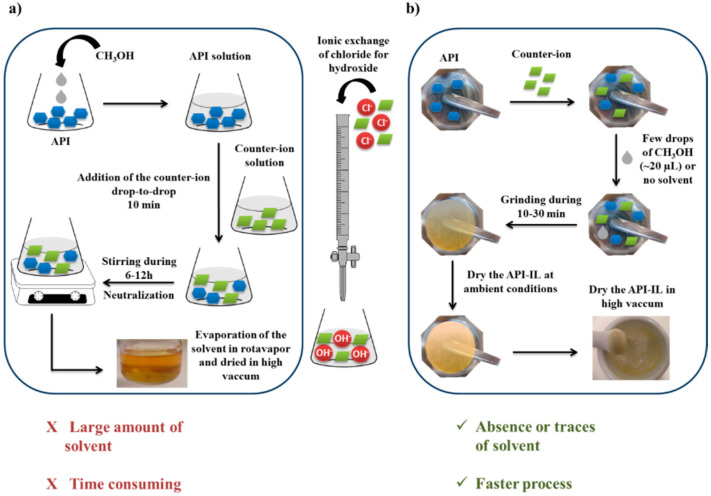
Schematic representation of (**a**) the traditional method and (**b**) new method. In the middle, the ionic exchange process, which is common in both strategies, is presented [46].

A strict comparison was made between the traditional solvent synthetic process and the mechanochemical techniques. An assessment between the two methods can be seen in Figure 5. The products obtained using both techniques were accurately characterized using ^1^H- and ^13^C-NMR spectroscopy, as well as differential scanning calorimetry (DSC). The purity of all compounds was ascertained by high-resolution MS, except in the case of (GBP) (gly) salt, for which an elemental analysis was performed because it was not possible to detect the glycolate anion owing to its low molecular weight (Table 2).

Mechanochemistry has proved to be a very promising synthetic strategy and a viable, efficient, and green alternative for the preparation of pharmaceutical ionic liquids. The results compare both the new and the classical approach and clearly show the advantages of the new method. This new technique is faster, solvent free, reproducible, selective, and leads to higher yields.

### 2.2. Sulfoxides: Robust Synthons in Co-Crystallization

A few years ago, Bernstein and his collaborators controversially established that diphenyl sulfoxide compounds have no tendency to form co-crystals [47]. Recently, Lawrence et al. [48] questioned this conclusion and studied the type of interactions present in supramolecular packings involving sulfoxide and sulfone compounds. They demonstrated that sulfoxide is a powerful hydrogen bond acceptor (N–H···O=S) for a variety of compounds with NH functional groups.

A significant number of drug molecules has sulfoxide groups, leading us to the study of two sulfonamides antibiotics, both highly insoluble, dapsone (DAP) and sulfadimethoxine (SDM), in order to improve their solubility and prove the possibility of expanding the pool of sulfoxide supramolecular interactions [49].

#### 2.2.1. Dapsone Co-Crystals

Dapsone (DAP) is used in the treatment of leprosy and pneumonia but also displays neuro-protective and anti-epileptic effects [49]. So far, no polymorphic forms are known [50], while some multicomponent derivatives were reported [50,51,52].

**Figure 6:**
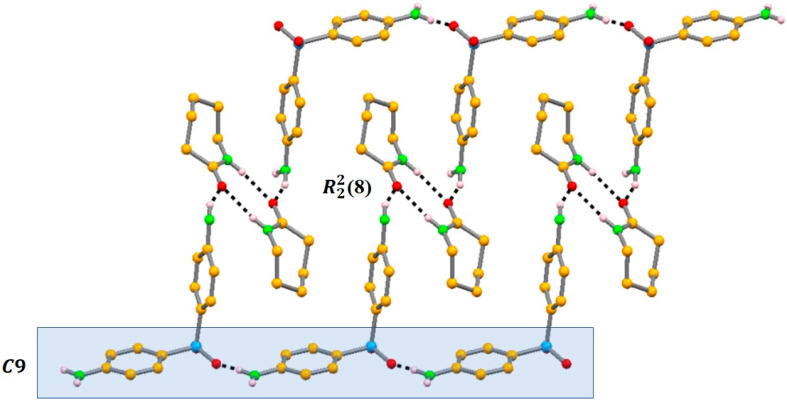
Crystalline packing of DAP:CAPRO: view along *c* showing the R^2^_2_(8) formed between CAPRO molecules and the capping by DAP molecules, supporting the chain of DAP molecules along *a* [49].

We succeeded in obtaining two cocrystals, one with caprolactam (CAPRO) and another with 4,4′-bipyridine (BIP). The DAP supramolecular arrangement is based on strong NH_NH2_···O_SO2_ hydrogen bonds, and some of them are disrupted in both co-crystals. In the first, DAP:CAPRO, one of the amine groups of DAP interacts preferentially with the amide moiety of CAPRO, N_NH2_···O_CONH_ interactions. DAP molecules still maintain one of the usual N–H···O_SO2_ hydrogen bonds between themselves, forming a one-dimensional chain based on C(9) synthons (Figure 6). In 2:1 (DAP:PYR), one of the DAP amine moieties interacts with the pyridine nitrogen atom (N_NH2_···N_pyridine_), again breaking the prevailing DAP–DAP interactions [49].

Having changed the supramolecular arrangement, in particular the breaking of the strong NH_NH2_···O_SO2_ hydrogen bond, we expected our products to be more soluble than DAP in aqueous solutions, but preliminary qualitative tests showed us that, unfortunately, we were wrong, as the solubility was not improved.

#### 2.2.2. Sulfadimethoxine: Learning about Synthon Competition

In order to continue our study on the specific interactions in molecules with sulfone moieties, we turned to sulfonamides, a family of antibacterial drugs. These pharmaceuticals were one of the first antibiotics to be used systematically, but bacteria soon developed resistance to them [53].

In co-crystallization works with compounds of the sulfonamide family [48,54,55] it has been observed that the sulfone moiety is a good hydrogen bond acceptor, due to the highly polar nature of the sulfur−oxygen bond. Grant et al. [56] verified that the amide proton is the best donor for sulfonamide hydrogen bonding and that the carbonyl, sulfanyl, and activated aromatic nitrogen groups are also good acceptors. In a review about sulfonamide salts, it is stated that the deprotonated amide nitrogen atom acts as the best acceptor [54]. These results highlight the number of hydrogen bonding possibilities and that the synthon competition is great. A crystal engineering overview on the effect of co-crystalization on sulfacetamide solubility modulation, published by Nangia et al. [57], also highlights this variety of interactions and synthons.

**Figure 7:**
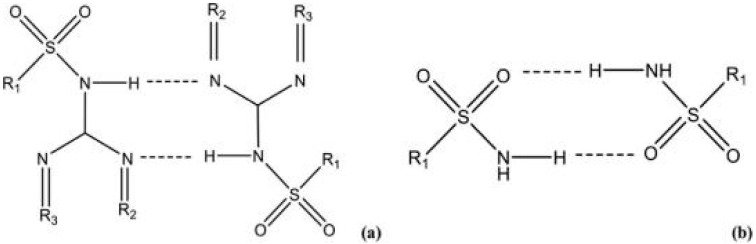
Pyrazine-substituted benzenesulfonamides R^2^_2_(8) synthons: (**a**) based on N−H_sulfonamide_···N_imine_ hydrogen bonds (Type I); (**b**) based on N−H_amide_···O_sulfone_ hydrogen bonds (Type II) [58].

We chose to work with sulfadimethoxine (SDM), and before attempting to prepare new crystal forms involving SDM, a Cambridge Structural Database (CSD) [50] search of synthons on pyridine-substituted benzenesulfonamides. The results obtained show that the most common synthon is the C(8) chain involving the N−H_terminal amine_···O_sulfone_ hydrogen bond, followed by an R^2^_2_(8) homosynthon, which can be obtained in two different ways: One making use of two N−H_amide_···N_imine_ hydrogen bonds (Type I) (Figure 7a) and the other by two N−H_sulfonamide_···O_sulfone_ hydrogen bonds (Type II) (Figure 7b) [58]. This way, we were able to select the best co-formers and we succeeded in obtaining eight new pure multicomponent crystal forms, using LAG.

In these new forms, the R^2^_2_(8) (Type II) synthon prevailing in the SDM crystal structure is always disrupted, while the chains remain unaffected, except in the presence of water molecules. As examples, we present and discuss here the case of the cocrystal with isonicotinamide (SDM:ISO) and the salt with piperazine (SDM:PIP). SDM is acidic enough to transfer the amide proton to PIP but not to ISO molecules.

**Figure 8:**
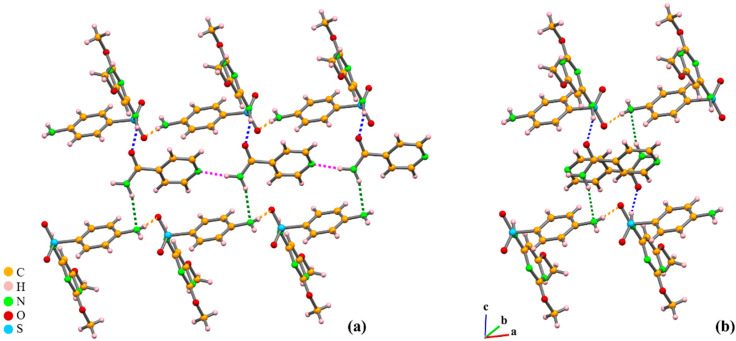
Crystal packing of SDM:ISO cocrystal: (**a**) in a view along the b-axis, with linear SDM chains interacting with two antiparallel ISO chains; (**b**) detail depicting the N−H_terminal amine_ (SDM)···O_sulfone_ (SDM) (yellow), N−H_sulfonamide_ (SDM)···O(ISO) (blue), and N−H_amide_ (ISO)···N_terminal amine_ (SDM) (green) interactions [58].

In SDM:ISO, the amide nitrogen, N_sulfonamide_, forms hydrogen bonds with the best acceptor of the co-former molecule, the acetamide oxygen. The C (8) synthon, formed by the terminal NH_2_ and the sulfone oxygen, is maintained (Figure 8).

**Figure 9:**
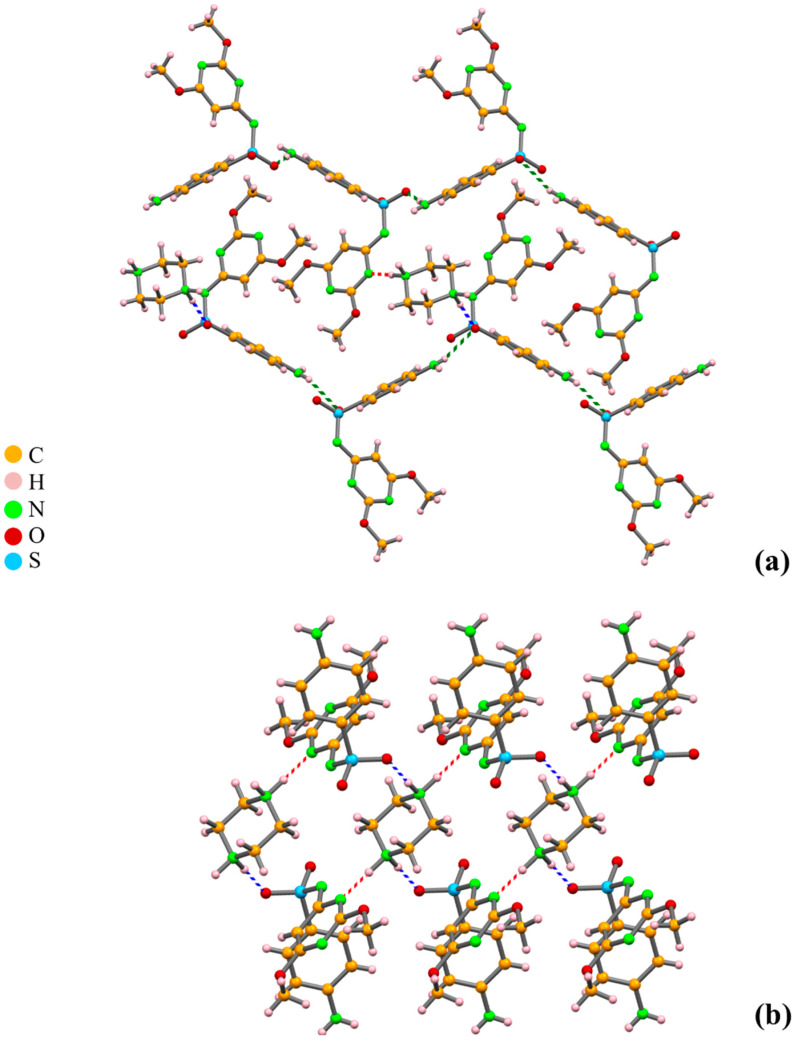
Crystal packing of the molecular salt SDM PIP: (**a**) view along *a* of SDM zigzag chains linked by PIP; (**b**) R^4^_4_(22) synthon formed by ^+^N−H_PIP_···O_sulfone_ − blue and ^+^N−H_PIP_···N_pyrazine ring_ − red) hydrogen bonds, displaying the full use of PIP^2+^ donor capacity [58].

In SDM:PIP salt, in which the best proton donor disappears due to the deprotonation of SDM, the N−H_terminal amine_···O_sulfone_ synthon C(8) is observed like in the cocrystal, but the amide moiety is not involved in supramolecular interactions. Salts also present hydrogen bonds between the protonated N of the conformer, in this case PIP, and the N atom of the sulfonamide group, but these charge-assisted interactions can also involve as an acceptor one of the oxygen atoms of the sulfonyl group as well as the N_pyrazine_ atom (Figure 9).

Preliminary water solubility tests revealed that all the compounds obtained are more soluble than the pure sulphonamide compound, the most soluble being the salts, as expected. 

### 2.3. Discerning Co-Crystals from Salts by X-ray Diffraction and Solid-State NMR 

In the crystal engineering community, it is well known that sometimes it is rather difficult to know if we are dealing with co-crystals or molecular salts, in particular due to the salt–co-crystal continuum [59]. To tackle this issue, we decided to study some co-crystals and salts with the two techniques that allow their full characterization: X-ray diffraction and solid-state NMR (SSNMR). The latter, well established in pharma science, has been used as a complementary technique to probe the short-range local structure, helping the positioning of light atoms, such as hydrogens [60,61,62,63], and in particular to study packing interactions, such as hydrogen bonds (HBs), and even establish their relative strength [62,64].

For this, we selected two APIs, azelaic acid (AA) and adamantilamine (ADA), so that we could study both a carboxylic derivative and an amine one.

#### 2.3.1. Azelaic Acid

AA is used to treat skin diseases, such as acne and rosacea. Usually, it is incorporated in creams or gels (20% and 15%, respectively) using alcohols as formulation solvents, due to its high insolubility in aqueous solutions. This can cause dehydration and seems to promote some instability of AA at room temperature in the lipid layer of the skin; thus, increasing the aqueous solubility of this compound is very important [65]. 

In the last decade, different multicomponent crystalline forms of AA were published, but mainly using solution as the main synthetic technique [66,67,68].

**Figure 10:**
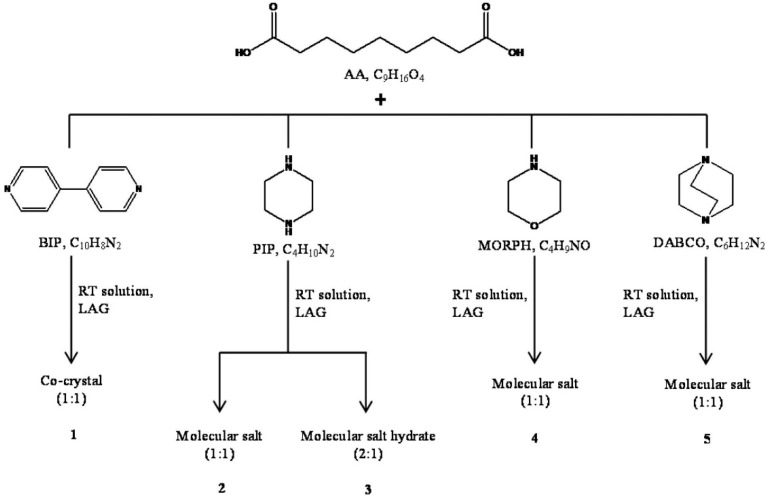
Experimental conditions and products obtained in the reactions between AA and BIP, PIP, MORPH, and DABCO [64].

In our laboratory, we synthetized by mechanochemistry (LAG) five new forms: (i) a co-crystal with 4,4′-bipyridine (AA:BIP); (ii) two molecular salts with piperazine, an anhydrous and a hydrated one (AA:PIP; AA:PIP:W); and (iii) two anhydrous molecular salts with morpholine (AA:MORPH) and 1,4-diazobicyclo [2.2.2]octane (AA:DABCO) (Figure 10).

As mentioned, their structural characterization was performed using single crystal X-ray diffraction and SSNMR supported by GIPAW-DFT calculations [69].

**Figure 11:**
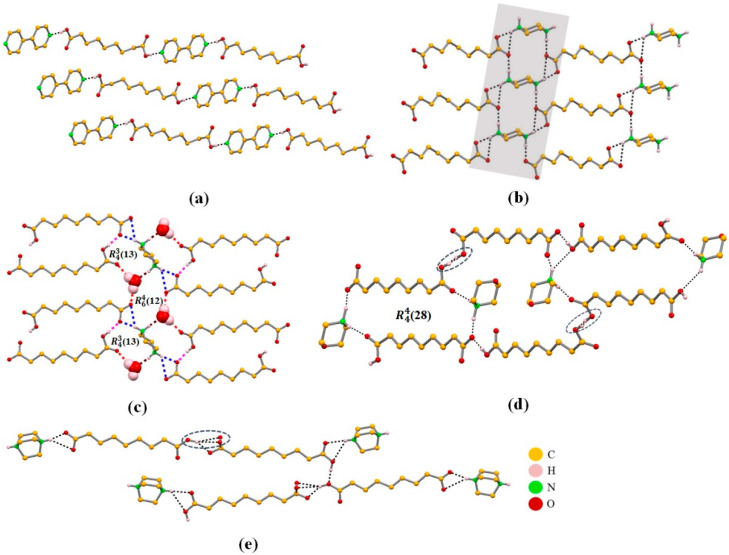
Packing diagrams showing the hydrogen bond OH_COOH_···N established in the co-crystal AA:BIP (**a**) and the charge-assisted interaction ^+^N−H···O_COO-_ in the molecular salts AA:PIP (**b**), AA:PIP: water (**c**), AA: MORPH (**d**), and AA:DABCO (**e**) [64].

Crystalline packing of all compounds shows that the typical R^2^_2_(8) homosynthon (carboxylic···carboxylic) present in the two polymorphs of AA is disrupted [50]. The cause of this is the preferential formation of the OH_COOH_···N hydrogen bond in the co-crystal, and the charged assisted ^+^N−H···O_COO-_ hydrogen bond in the molecular salts. These interactions overcome the formation of OH_COOH_···O_COO−_ synthons and stabilize the AA:co-former structure (Figure 11).

Unambiguous assignment of the salt type of compound was achieved by both ^1^H MAS NMR and ^13^C CPMAS spectra, detecting respectively a strong N−H···O hydrogen bond and the typical spectrum of COOH in COO− groups. 

Thermal and room stability studies were conducted in all the new forms, and we could conclude that their behavior strongly depends on the supramolecular structure and on the strength of the hydrogen bond network.

The new AA:PIP molecular salt presented a higher value of solubility in water (AA, 10 mg/7.5 mL of water against 10 mg/0.5 mL of water for the salt), proving to be a safer alternative to the use of alcoholic solutions in the final formulations. This opens a new perspective for the use of these salts in the market.

#### 2.3.2. Adamantylamine

Adamantylamine (ADA) is a low-affinity noncompetitive *N*-methyl-d-aspartate receptor antagonist widely used in the treatment of Parkinson’s disease [70,71]. There are also references to its use in the treatment of influenza (H5N1) as well as depression, alone or combined with other drugs [72,73]. ADA-free base has a very low water solubility (1.03 mg/mL) and is currently administrated in hydrochloride form (ADA·HCl), in Europe and the USA [38]. However, it was observed to be associated with some toxic effects on the central nervous system [74], and a new salt (ADA sulfate) has been tested, revealing some advantages, such as a slow concentration increase in blood plasma. This effect allowed the possibility to increase the daily dosage for treating long-term Parkinson’s disease [75].

**Figure 12:**
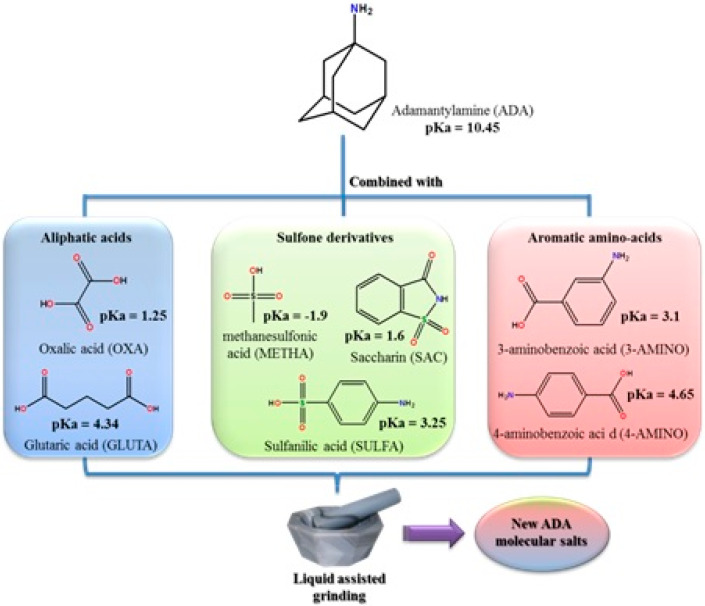
General experimental conditions and products obtained in the reactions between adamantylamine and aliphatic acids, sulfone derivatives, and aromatic acids [76].

In our lab, we decided to mechanochemically synthesize some other ADA salts, using generally recognized as safe (GRAS) co-formers (Figure 12).

From this pool, we were able to isolate in a pure form seven salts: ADA:OXA, ADA:METHA, ADA:SAC, ADA:SULFA, ADA:4-AMINO, ADA:GLUTA, and ADA:3-AMINO. To carefully characterize these compounds, complementary techniques were combined, such as single crystal and powder X-ray diffraction, ^13^C- and ^15^N-solid-state-NMR, and FTIR-ATR. 

**Figure 13:**
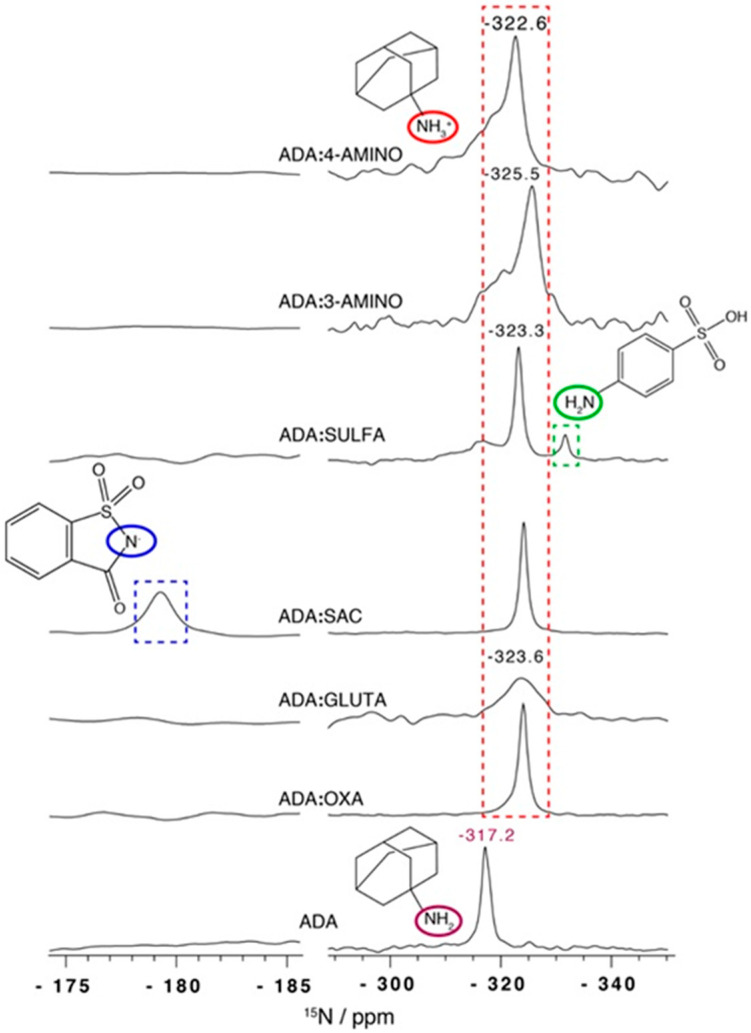
^15^N CPMAS NMR spectra for all salts. Red, blue, and green rectangles highlight the ^15^N CS associated with ADA protonated amine, saccarin and sulfanilic acid, respectively [76].

^15^N-SSNMR analysis was extremely helpful, as it was easy to assign the protonation of the ADA amine moiety (NH_3_^+^), a shift of 5 to 8 ppm when compared with NH_2_ of pristine ADA (Figure 13).

**Figure 14:**
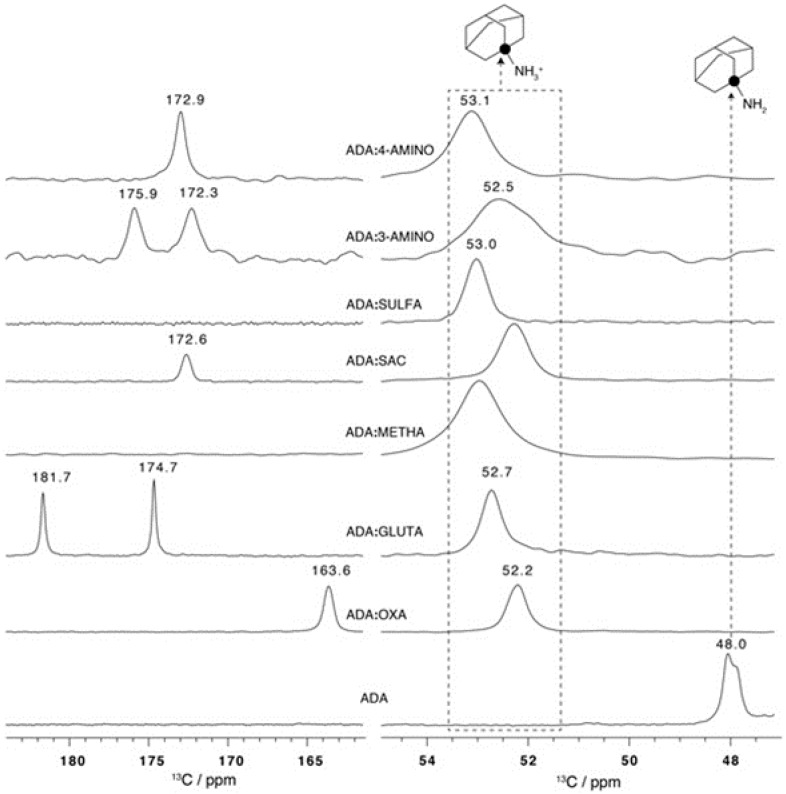
^13^C CPMAS NMR spectra for all salts, showing the CS range for C1 (right side) and carboxylic/carboxylate resonances (left side). Filled circles in the ADA structure indicate the position of the C1 quaternary carbon [76].

Salt formation was also confirmed through the analysis of ^13^C CPMAS NMR, with a special emphasis on the chemical shift variation of the quaternary carbon directly attached to the amine of ADA, which is very sensitive to the protonation state of this amine group (Figure 14).

**Figure 15:**
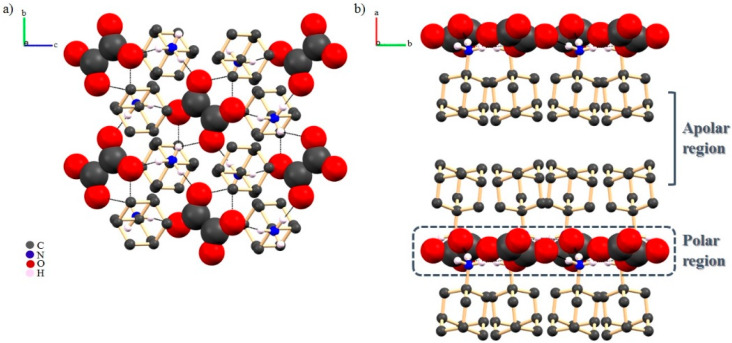
(**a**) view along *a* evidencing N-H^+^(ADA)···O^-^(OXA) interactions in a mosaic-like fashion mode; (**b**) view along *b* showing the lamellar effect. Aliphatic hydrogen atoms were omitted for clarity [76].

In all molecular salts, the crystal packing is supported on a common synthon, a ^+^N−H(ADA)···O−(co-former) charge-assisted hydrogen bond. Even though the supramolecular arrangement is supported in the same type of interaction, we observed that depending on the size and functional groups of the co-former, different overall structures were obtained. For small co-formers, such as OXA or METHA, a lamellar-like packing of alternated polar and apolar domains was obtained (Figure 15).

**Figure 16:**
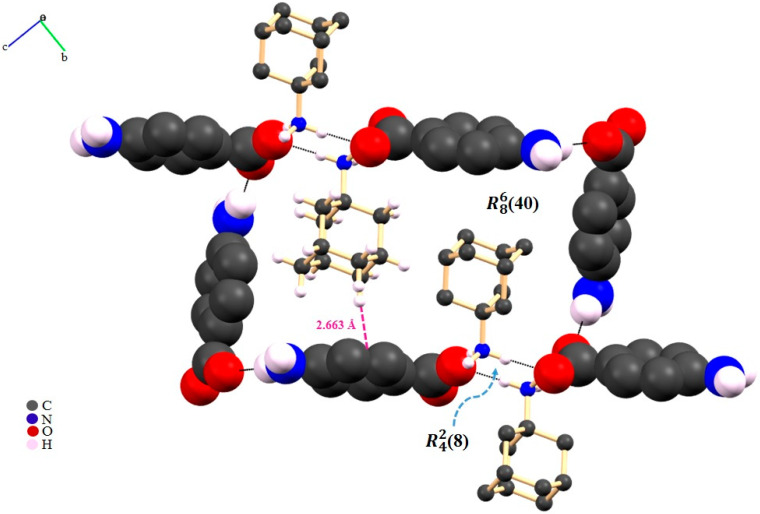
Packing interactions highlighting the R^2^_2_(8) synthon between ADA and 4-AMINO molecules (N-H^+^_(ADA)_···O^−^_(4-AMINO)_ HB). The presence of the N-H···_(4-AMINO)_···O^-^_(4-AMINO)_ HB interaction allows the R^6^_8_(40) synthon, where ADA aliphatic domains face each other. Some aliphatic hydrogen atoms were omitted for clarity [76].

For the other co-formers, as in the case of 4-Amino, a zeolite-like packing was attained (Figure 16).

All of the salts presented improved physicochemical properties when compared to ADA-free base, but only ADA:METHA and ADA:GLUTA performed better than the commercial salt distributed in the market (Table 3).

## 3. Conclusions

As mentioned, this short overview presents and highlights the work performed by the Lisbon group on the mechanochemical synthesis of active pharmaceutical ingredient (API) multicomponent compounds. This was not an exhaustive presentation and we decided to choose from our work some specific areas: systematic studies on gabapentin polymorphism, crystal engineering based studies exploring new functional groups, and the use of complementarity techniques to discern between co-crystals and molecular salts. 

The results obtained on gabapentin polymorphic screening, using mechanochemistry, and the method by which we were able to induce and control its different polymorphic forms using RTILs are completely new. In this approach, we were also able to isolate and maintain the different forms for 4 months, if they were kept soaked. Additionally, we produced for the first time several ionic liquids bearing the drug, avoiding gabapentin polymorphic behavior.

As an application of crystal engineering principles, we presented our work using sulfoxides as robust synthons, analyzing the supramolecular arrangement obtained in multicomponent crystal forms of dapsone and sulfadimethoxine. We correlated their crystal packing with their physicochemical properties, such as thermal and humidity stability and solubility. All the SDM salts presented a higher solubility than pristine sulfadimethoxine.

To distinguish cocrystals from salts using a unique structural characterization technique is sometimes impossible. We presented the results obtained for azelaic acid and adamantylamine; simultaneously, X-ray diffraction, SSNMR, and DFT calculations were used. 

We also want to highlight that mechanochemistry has proved to be a very promising synthetic technique, and that, when comparing the results obtained in the preparation of pharmaceutical ionic liquids, the new technique proved to be faster, solvent free, reproducible, selective, and leading to higher yields.

## Figures and Tables

**Table 1:** Gabapentine polymorphic forms identified by single crystal XRD data [44].

Entry	RTIL in Methanol	RT	RT→LT→RT	RT→HT→RT	RT→HT→LT→RT
1	Control Sample^a^	Form II	Form II	Form II	Form II
2	C_4_mimN(CF_3_SO_2_)_2_	Form II	Form IV	Form III	Form III
3	C_4_mimBF_4_	Form II	Form II + III	Form II	Form II + IV
4	C_6_mimBF_4_	Form II	Form III	Form II + IV	Form IV
5	C_4_mimBF_4_ + C_6_mimBF_4_	Form II	Form III	Form IV	Form IV
6	C_4_mimN(CF_3_SO_2_)_2_ + C_6_mimN(CF_3_SO_2_)_2_	Form II + III	Form II + III + IV	Form III	Form IV
7	C_6_mimN(CF_3_SO_2_)_2_ + C_6_mimBF_4_	Form II	Form II	Form II	Form II

^a^This control sample does not contain RTIL and was used for comparison purposes with respect to the assays containing RTILs.

**Table 2:** Physical state, yield, thermal properties (*T_g_*), and reaction time obtained for all compounds in each synthetic method [46].

	Acid-base Neutralization (Traditional Method)				Mechanochemistry (New Method)			
Compound Name ^a^	Physical State at RT	Yield (%)	*T_g_* (°C)	Reaction Time (h)	Physical State at RT	Yield (%)	*T_g_* (°C)	Reaction Time (min)
(EMIM)(GBP)	Transparent liquid	96.5	−70.9	15	Transparent liquid	97.7	−72.2	180^b^
(choline)(GBP)	Transparent viscous liquid	98.5	−59.2	15	Transparent viscous liquid	99.0	−59.0	180^b^
(GBP)(D-glu)	Orange viscous liquid	92.5	−48.6	10	Orange viscous liquid	95.2	−44.0	30
(GBP)(gly)	Transparent liquid	97.2	−56.2	10	Transparent liquid	99.3	−49.1	15
(TMG)(L-glut)	Light yellow liquid	93.7	−36.2	6	Light yellow liquid	97.0	−38.9	10
(DBU)(L-glut)	Yellow liquid	98.4	−39.5	6	Yellow liquid	99.5	−44.5	10

^a^ GBP: gabapentine/solid; EMIN—ethyl-methylimidazolium/solid; D-glu: D-gluconic acid/solid; gly: glycolic acid/solid; TMG: tetramethylguanidine/liquid; L-glut: L-glutamic acid/solid; DBU: 1,8-diazabicyclo[5.4.0]undec-7-ene/liquid. ^b^The time considered is the total time starting from the ionic exchange process and the concentration of the reagent (to eliminate the solvent excess) until the end of the reaction itself (grinding both starting materials). Considering the grinding process, only 30 min were required to finish the reaction.

**Table 3:** Solubility, thermal stability, in ADA, ADA∙HCl, and molecular salts [76].

Compound	Solubility (mg/mL)	Melting Point (°C)	Melting Point Conformers (°C)
ADA	1.03	206–208	-
ADA∙HCl	50	300	-
ADA:OXA (I)	0.81	223	189–191
ADA:GLUTA (II)	58.8	183	95–98
ADA:METHA (V)	200	153.9 ^a^	17–19
ADA:SAC (VI)	14.3	242.6	228
ADA:SULFA (VII)	5.6	347.3 ^a^	288
ADA:3-AMINO (VIII)	9.1	244.2	178–180
ADA:4-AMINO (IX)	0.96	245.9	187–189

^a^ Melting point determined by the glass capillary method and by DSC-TGA.
