# Addendum: da Silva, J.L.F.; et al. The Lisbon Supramolecular Green Story: Mechanochemistry towards New Forms of Pharmaceuticals. *Molecules* 2020, *25*, 2705

**DOI:** 10.3390/molecules26020419

**Published:** 2021-01-14

**Authors:** João Luís Ferreira da Silva, M. Fátima Minas da Piedade, Vânia André, Sofia Domingos, Inês C. B. Martins, M. Teresa Duarte

**Affiliations:** 1 Centro de Química Estrutural, Instituto Superior Técnico, Universidade de Lisboa, Avenida Rovisco Pais, 1049-001 Lisboa, Portugal; joao.luis@ist.utl.pt (J.L.F.d.S.); mdpiedade@fc.ul.pt (M.F.M.d.P.); vaniandre@tecnico.ulisboa.pt (V.A.); sofiadomingos@ff.ulisboa.pt (S.D.); ines.martins@tecnico.ulisboa.pt (I.C.B.M.); 2 Departamento de Engenharia Química, Instituto Superior Técnico, Universidade de Lisboa, Avenida Rovisco Pais, 1049-001 Lisboa, Portugal; 3 Departamento de Química e Bioquímica, Faculdade de Ciencias, Universidade de Lisboa, 1649-016 Lisboa, Portugal; 4 Associação do Instituto Superior Técnico para a Investigação e Desenvolvimento (IST-ID), Av. Rovisco Pais, 1049-003 Lisboa, Portugal

The authors wish to add funding project UIDB/00100/2020 into the Funding section of this paper [1]. The corrected funding is provided below:
